# Bacteriologically-confirmed pulmonary tuberculosis in an Ethiopian prison: Prevalence from screening of entrant and resident prisoners

**DOI:** 10.1371/journal.pone.0226160

**Published:** 2019-12-12

**Authors:** Eliyas Tsegaye Sahle, Jill Blumenthal, Sonia Jain, Shelly Sun, Jason Young, Tsegahun Manyazewal, Habtamu Woldeamanuel, Lemma Teferra, Beniam Feleke, Olivier Vandenberg, Zilma Rey, Melissa Briggs-Hagen, Richard Haubrich, Wondwossen Amogne, John Allen McCutchan

**Affiliations:** 1 ADDIS-VP Project, Ethiopian Public Health Association, Addis Ababa, Ethiopia; 2 École de Santé Publique, Université Libre de Bruxelles, Brussels, Belgium; 3 University of California San Diego, San Diego, California, United States of America; 4 Addis Ababa University, College of Health Sciences, Addis Ababa, Ethiopia; 5 Ethiopian Federal Prison Administration, Addis Ababa, Ethiopia; 6 Centers for Disease Control and Prevention, Addis Ababa, Ethiopia; 7 Environmental and Occupational Health Research Centre (CRSET), School of Public Health, Université Libre de Bruxelles, Brussels, Belgium; 8 Division of Infection & Immunity, University College London, London, United Kingdom; 9 Centers for Disease Control and Prevention, Atlanta, Georgia, United States of America; 10 Gilead Sciences, Foster City, California, United States of America; Jamia Hamdard, INDIA

## Abstract

**Background:**

Pulmonary Tuberculosis (PTB) is a major health problem in prisons. Multiple studies of TB in regional Ethiopian prisons have assessed prevalence and risk factors but have not examined recently implemented screening programs for TB in prisons. This study compares bacteriologically-confirmed PTB (BC-PTB) prevalence in prison entrants versus residents and identifies risk factors for PTB in Kality prison, a large federal Ethiopian prison located in Addis Ababa, through a study of an enhanced TB screening program.

**Methods:**

Participating prisoners (n = 13,803) consisted of 8,228 entrants screened continuously and 5,575 residents screened in two cross-sectional waves for PTB symptoms, demographics, TB risk factors, and medical history. Participants reporting at least one symptom of PTB were asked to produce sputum which was examined by microscopy for acid-fast bacilli, Xpert MTB/RIF assay and MGIT liquid culture. Prevalence of BC-PTB, defined as evidence of *Mycobacterium tuberculosis* (MTB) in sputum by the above methods, was compared in entrants and residents for the study. Descriptive analysis of prevalence was followed by bivariate and multivariate analyses of risk factors.

**Results:**

Prisoners were mainly male (86%), young (median age 26 years) and literate (89%). Prevalence of TB symptoms by screening was 17% (2,334/13,803) with rates in residents >5-fold higher than entrants. Prevalence of BC-PTB detected by screening in participating prisoners was 0.16% (22/13,803). Prevalence in residents increased in the second resident screening compared to the first (R1 = 0.10% and R2 = 0.39%, p = 0.027), but remained higher than in entrants (4.3-fold higher during R1 and 3.1-fold higher during R2). Drug resistance (DR) was found in 38% (5/13) of culture-isolated MTB. Risk factors including being ever diagnosed with TB, history of TB contact and low Body Mass Index (BMI) (<18.5) were significantly associated with BC-PTB (*p*<0.05).

**Conclusions:**

BC-PTB prevalence was strikingly lower than previously reported from other Ethiopian prisons. PTB appears to be transmitted within this prison based on its higher prevalence in residents than in entrants. Whether a sustained program of PTB screening of entrants and/or residents reduces prevalence of PTB in prisons is not clear from this study, but our findings suggest that resources should be prioritized to resident, rather than entrant, screening due to higher BC-PTB prevalence. Detection of multi- and mono-DR TB in both entrant and resident prisoners warrants regular screening for active TB and adoption of methods to detect drug resistance.

## Introduction

Tuberculosis (TB) is a major cause of morbidity and mortality in low-income countries globally, killing an estimated 1.1 million HIV-negative and 0.4 million HIV-positive people globally **[1]**. Ninety-nine percent of deaths occur in developing countries, mostly in Sub-Saharan Africa and South East Asia **[2]**. TB costs the average patient three or four months of lost earnings (representing up to 30 percent of annual household income) **[3]**. Globally, Ethiopia is the 7^th^ highest in TB burden and 15^th^ with new cases of MDR-TB each year among the 27 countries with highest number of cases of multi-drug resistant (MDR) TB **[4]**. Although TB prevalence in Ethiopia has steadily declined, prevalence of clinically diagnosed pulmonary TB (PTB) remains high at 211 per 100,000 populations **[2]**.

An estimated 86,610 inmates were incarcerated in 119 prisons in Ethiopia in 2012 **[5]**. The TB problem in many prisons is exacerbated by: (a) conditions that increase the risk of reactivation and transmission such as malnutrition, physical and psychological stressors, overcrowding, poor ventilation, and HIV infections and (b) limited access to and poor quality of medical care **[6–10]**. In addition, transfers of inmates within and among prisons may contribute to ongoing disease transmission by increasing exposures of contagious pulmonary TB prisoners to uninfected prisoners **[11,12]**.

Prevalence studies from Sub-Saharan African prisons suggest that 0.7% to 5.8% prisoners have active TB **[8,11,12]**. Estimated PTB prevalence rates in residents of four Ethiopian regional prisons have been high, ranging from 458 to 1,913/100,000 prisoners. None of these studies have systematically screened for PTB in entrants to the prison. To assess TB prevalence and evidence of intramural transmission or reactivation in the central Ethiopian federal prison, we examined data from a screening program which we enhanced by adding laboratory and radiographic diagnostic capacity. We report the prevalence of bacteriologically-confirmed (BC)-PTB and associated risk factors among entrant and resident prisoners.

## Methods

### Study design

Two independent, cross-sectional surveys screened both resident (R) and newly-admitted, entrant (E) prisoners for symptoms of PTB and HIV from 8/2014 through 11/2016 in two waves: (R1&E1 = 8/2014 through 3/2015) and (R2&E2 = 11/2015 to 11/2016). The second wave was more prolonged due to delayed approval to add additional testing. Entrants who were screened in the gap between the two waves of screenings (EG = 3/2015 to 11/2016) are tabulated separately.

### Study site

Kality Federal Prison is located 15 kilometers from central Addis Ababa, Ethiopia. It provides long-term incarceration, shorter-term detention during trials, and temporary holding of prisoners before transfer to other federal prisons. It consists of a central high-security area divided into eight zones and an adjacent lower security area that contains medical clinics that provide management of HIV, TB, and syphilis, a 40-bed TB and general medical ward, and a hospital containing a well-equipped laboratory, a digital x-ray facility and two inpatient wards with a total of 58 beds.

On average about 60 new prisoners enter the prison daily and the average resident prisoner census is about 4,500. Full-time medical staff at Kality consists of 3 physicians, 9 health officers (physician’s assistants), 40 nurses, 3 radiography technicians and a consulting radiologist, and 6 laboratory technicians. Clinics specific for TB and HIV manage all prisoners with these diagnoses, including those referred from other prisons.

### Recruitment and screening

Potential participants were recruited by prison medical staff either during health screening at entry (entrants) or at group meetings within their prison zones (residents). Those who qualified met criteria listed below:

*Inclusion Criteria*: a) Men or women age 18 years or older, b) either newly entering the prison or a current resident of the prison; and c) able and willing to provide informed consent.

*Exclusion Criteria*: a) Cognitive impairment from conditions such as severe illness or injury, developmental retardation, or psychiatric illness that preclude informed consent or safely participating in study procedures; and (b) for residents, participation in screening at entry within the past 30 days. Prisoners with established diagnoses of PTB were screened by questionnaire, but did not have sputum examinations or study- related chest x-rays.

### Clinical data collection

Case report forms (CRFs) consisted of a structured questionnaire comprised of demographic data, clinical symptoms and medical history (including diagnoses, treatment and risk factors for TB and HIV), laboratory data, and radiologic findings. Data were collected by trained health care workers and recorded on paper forms labeled only with a unique participant identification number. To assess for active TB symptoms, we utilized the standardized World Health Organization (WHO) TB symptom screening tool **[13]**. Prisoners with possible TB symptoms were defined by their reporting of least 1 out of 5 symptoms: cough of > 2 weeks duration (or any duration for known HIV-positive), night sweats, weight loss, fever or hemoptysis. Meeting these criteria triggered isolation, and laboratory and chest x-ray (CXR) investigation for active PTB, if logistically possible. Height and body weight were measured on a height stand and electronic digital scale, respectively.

### Specimen collection, processing, and testing

The standard evaluation of prisoners with suspected PTB in Ethiopia includes three sputums for acid-fast bacilli (AFB) microscopy and CXR as available. The Kality Prison laboratory performed 3 AFB smears on sputum collected using the “spot-morning-spot” method **[14]**. Specimens were visually assessed for quality (mucoid to muco-purulent), examined for AFB microscopy in the Kality laboratory, stored at 2–8 ^o^C. The best quality sputum per prisoner was selected for the Xpert assay which detects *Mycobacterium tuberculosis* (MTB) DNA and mutations associated with rifampicin resistance (Cepheid, California, USA). For the first 8 months of the study, the Addis Ababa Regional Laboratory and thereafter the Kality laboratory performed the Xpert assay.

All microscopically positive (AFB +) and 10% of microscopically negative (AFB -) sputum samples of adequate quality and volume were submitted to the private, internationally-accredited International Clinical Laboratory (ICL). ICL performed liquid culture and growth-based drug sensitivity testing (DST) for five drugs [isoniazid (INH), rifampicin (RIF), ethambutol (ETB), pyrazinamide (PZA), and streptomycin (STM)] using the BACTEC MGIT960 system (Becton Dickinson, Maryland, USA). Rapid molecular DST for INH and RIF was performed with the GenoType MTBDRplus line probe assay (LPA) (Hain Lifescience GmbH, Germany).

In addition, CXR was performed on 1086 of 2334 (46.5%) prisoners who were TB symptom screen positive, but these results are not discussed in this paper. All consenting participants underwent HIV antibody screening where reactive tests were tested with a combination of serial rapid HIV tests according to the prevailing Ethiopian national HIV testing algorithm. ICL retested all HIV positive samples and 5% of randomly selected HIV negative samples by Abbott/Architect HIV Antigen/Antibody (Ag/Ab) assay for quality assurance.

**Fig 1:**
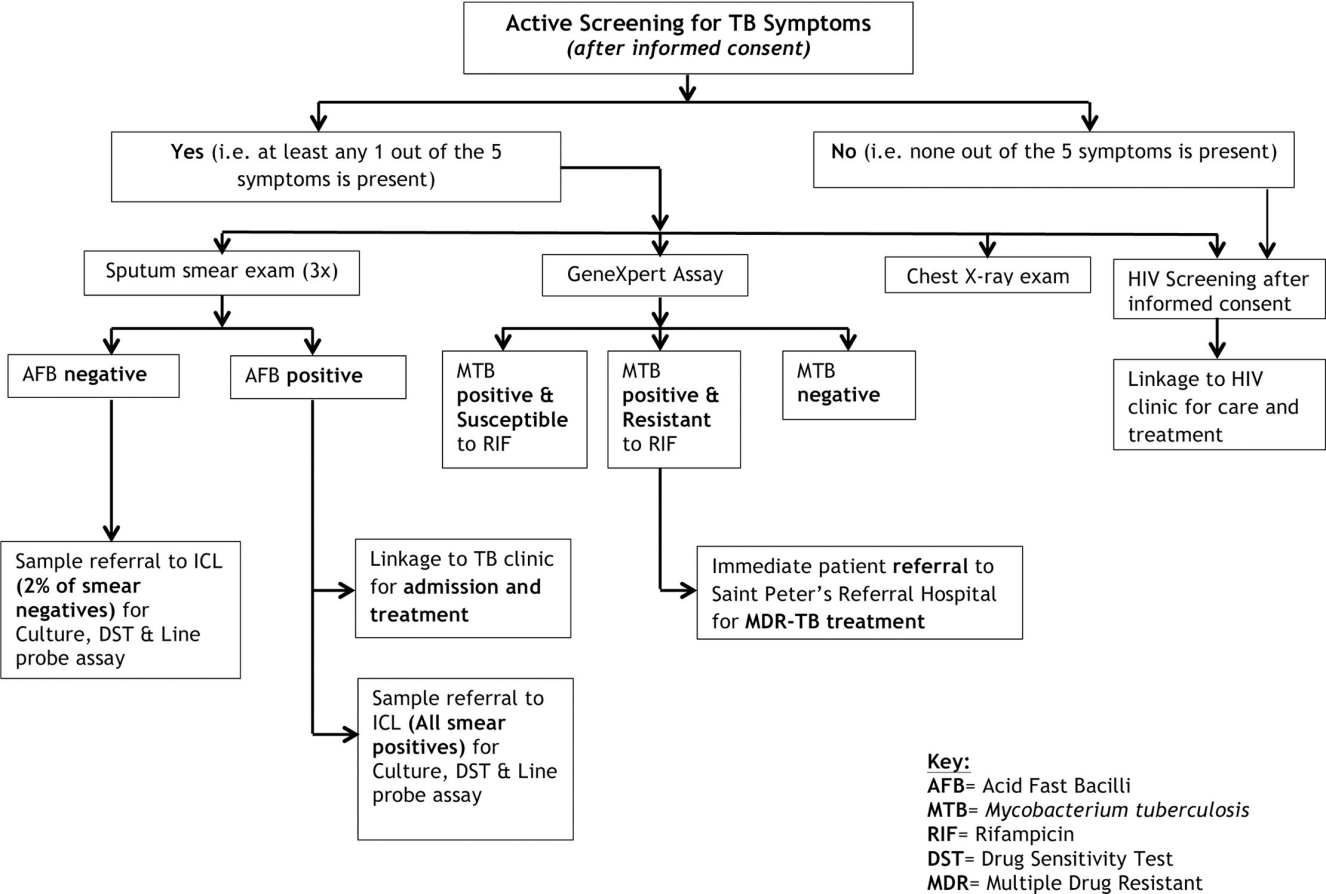
Work flow for TB screening.

A summary of the recruitment, TB symptom screening, additional diagnostic procedures, and referral for treatment that generated the study data shown is shown in Fig 1.

### Data collection and oversight

Data was collected using paper case report forms (CRFs) and directly entered into the University of California San Diego (UCSD)-based Open-Source Clinical Content Analysis and Management System (OCCAMS) for research data management by trained data entry clerks at Kality. The investigators reviewed regular reports of accumulating data provided by the UCSD-based statistical team. Data quality was internally assessed by complete auditing by the data manager and was externally assessed by the Centers for Disease Control and Prevention (CDC) three times and by an independent auditor two times during the study. Formal reporting of audits identified deficiencies and errors to which the study team responded with appropriate corrections and methodological adjustments.

### Definitions of terms and acronyms

In classifying PTB cases we utilized the following definitions the first of which differs from Ethiopian national guidelines by diagnosing PTB based on only one sputum positive for AFB **[14]**.

*Entrants (E)*: prisoners newly entering the prison
*Residents (R)*: prisoners who have been in prison for ≥30 days
*Bacteriologially-confirmed pulmonary* TB *(BC-PTB)*: diagnosis by AFB smear, nucleic acid detection (Xpert MTB/RIF) or liquid culture of sputum
*Mono-drug resistance (Mono-DR)*: resistance to one first-line anti-TB drug only
*Multi-drug resistance (MDR*): resistance to at least both INH and RIF

### Statistical analysis

Descriptive analyses were performed to study the socio-demographics and prevalence of PTB symptoms and BC-PTB by entrants (E1, E2, EG) and residents (R1, R2), separately and overall. Prevalence rates were compared between entrants and residents using Fisher’s exact test. Univariate associations between potential risk factors and BC-PTB were assessed using Fisher’s exact test for categorical variables and Wilcoxon Rank Sum test for continuous variables. Binary logistic regression was performed to identify risk factors which predict occurrence of BC-PTB controlled for others.

### Ethical review and consenting procedures

This study was conducted after protocol review and approval by the Ethiopian Federal Prison Administration, National Research Ethics Review Committee of the Ethiopian Ministry of Science and Technology, Internal and Scientific and Ethical Review Committee of the Ethiopian Public Health Association, and UCSD. The study was also reviewed in accordance with the Centers for Disease Control and Prevention (CDC) human research protection procedures and was determined to be research, but CDC investigators did not interact with human subjects or have access to identifiable data or specimens for research purposes.

Written approval for this research was obtained from the following ethics committees / IRBs: a) University of California San Diego, Human Research Protections Program, Approval number: 140109; b) Federal Democratic Republic of Ethiopia Ministry of Science and Technology, National Research Ethics Review Committee (NRERC), Approval number: 3-10/797106; and c) Ethiopian Public Health Association, Approval number: EPHA /00/1345014.

Recruiters who were trained in ethical conduct of research obtained written informed consent by providing information to groups of prisoners followed by individual interviews to answer answer questions and complete the screening questionnaires. The English language consent form was translated to Amharic, the national language of Ethiopia, and validated through back translation to English. Illiterate prisoners were read the consent document as part of the group consenting process, given time to ask questions individually with the recruiting medical personnel, and then signed their form with a witness to the process.

## Results

### Participant characteristics

The majority of the participants (both entrants and residents) were male (86%) and with median age of 26 years (range: 18–90; IQR: 21–33) (Table 1). The majority identified their ethnicity primarily as either Amhara (39%) or Oromo (24%). Eighty-nine percent were able to read and write in Amharic although most completed only elementary education or less (54%). Most had never married (61%), were from urban area (92%), and had no prior imprisonment (96%).

**Table 1:** Socio-demographic characteristics of 13,803 study participants.

Variable	Category	Value or n (%) (out of N = 13,803)
**Age**	Median (IQR)	26 (21–33)
**Gender**	Male	11,823 (85.7)
**Ethnicity**	Amhara	5,341 (38.7)
	Oromo	3,262 (23.6)
	Southern	2,872 (20.8)
	Tigre	1360 (9.9)
	Others	968 (7.0)
**Education**	None	1,564 (11.3)
	Elementary (0–8)	5,891 (42.7)
	Secondary (9–10)	3,253 (23.6)
	*Post-secondary	3,095 (22.4)
**Literacy**	Literate	12,258 (88.8)
	Illiterate	1,545 (11.2)
**Marital Status**	Never Married	8,470 (61.4)
	Married	4,938 (35.8)
	**Other	395 (2.8)
**Residence before prison**	Urban	12,698 (92.0)
	Rural	1,096 (7.9)
	Pastoral	6 (0.1)
**Prior Imprisonment**	Yes	536 (3.9)
	No	13,267 (96.1)

*Post-secondary: Preparatory (11–12), Diploma, Undergraduate, and Post-grad Masters/PhD

**Other: Divorce/Separated, Widow/Widower, and other types of relationships.

### PTB screening and prevalence

All 13,803 consenting participants were screened for PTB symptoms using the standardized WHO TB symptom screening tool. Of 13,803 prisoners screened for PTB symptoms, 2,334 (17%) reported at least one TB symptom. Among those with qualifying PTB symptoms, 1760/2334 (75.4%) reported cough of ≥2 weeks, 1,415 (60.6%) had fever, 333 (14.3%) hemoptysis, 1,374 (58.9%) had night sweats, 1,013 (43.4%) had weight loss and 91 (0.7%) were HIV+ with cough of any duration (Table 2). Prevalence of PTB symptoms by screening was consistently more than five-fold higher in residents than entrants: 5.4 (38%/7%) fold higher in R1 than E1, p<0.001; and 6.2 (31%/5%) fold higher in R2 than E2, p<0.001) (Table 2).

**Table 2:** Prevalence of PTB symptom screen and bacteriologically confirmed-PTB cases.

	n, (Prevalence in %)						p-value	
	E1 n = 1,569	E2 n = 3,389	EG n = 3,270	R1 n = 3,024	R2 n = 2,551	Total n = 13,803	E1 vs R1	E2 vs R2
**PTB Symptom +**	112 (7)	165 (5)	108 (3)	1152 (38)	797 (31)	2334 (17)	<0.001	<0.001
**Cough >2weeks**	74 (5)	116 (3)	70 (2)	832 (28)	668(26)	1760 (13)	<0.001	<0.001
**Fever**	53 (3)	122 (4)	75 (2)	670 (22)	495 (19)	1415 (10)	<0.001	<0.001
**Hemoptysis**	15 (1)	28 (1)	21 (1)	149 (5)	120 (5)	333 (2)	<0.001	<0.001
**Night sweats**	59 (4)	112 (3)	78 (2)	671 (22)	454 (18)	1374 (10)	<0.001	<0.001
**Weight loss**	47 (3)	85 (3)	40 (1)	482 (16)	360 (14)	1014 (7)	<0.001	<0.001
**HIV+ with current cough**	3 (0.19)	4 (0.12)	3 (0.09)	36 (1.2)	38 (1.5)	84 (0.61)	<0.001	<0.001
**Self-reported current TBTx**	2 (0.13)	1 (0.03)	6 (0.18)	10 (0.33)	14 (0.55)	33 (0.24)	0.240	<0.001
**Total cases of BC-PTB**	1 (0.06)	3 (0.09)	5 (0.15)	3 (0.10)	10 (0.39)	22 (0.16)	1	0.021

E = Entrants, R = Residents, EG = Entrants screened between E1 and E2 screening, BC-PTB = Bacteriologically-confirmed pulmonary tuberculosis, TBTx = TB treatment

The overall prevalence rate of BC-PTB by screening was 0.16% or 159/10^5^ (22/13,803) with rates of 0.11% or 109/10^5^ (9/8,228) in entrants and 0.23% or 233/10^5^ (13/5575) in residents. Four cases were identified among entrants for a prevalence of 0.06% or 64/10^5^ (1/1569) in E1 and 0.09% or 88/10^5^ (3/3389) in E2 screening periods. An additional 0.15% or 153/10^5^ (5/3,270) were found when entrants were screened in the gap between resident waves (EG). Thirteen cases were found in residents, and the prevalence of BC-PTB was nearly four-fold higher in R2 (0.39% or 10/2,551) than in R1 (0.10% or 3/3,024) screening periods.

### Diagnostics of bacteriologically-confirmed PTB cases

We identified a total of 22 BC-PTB cases, of which 18 (82%) were found by AFB smear microscopy. Of the 4 cases that were AFB smear negative, 3 were positive by culture only and 1 by culture and Xpert (Table 3). Culture was negative in 9 cases in which both AFB microscopy and Xpert were positive. Of 738 sputums submitted for Xpert testing, 19 were positive (Table 4). Xpert testing for MTB detection compared to AFB and culture (BC-TB excluding Xpert) had sensitivity of 85.7% (18/21) and specificity was 99.9% (785/786). Compared to results from 738 AFB smears, sensitivity was 100% (18/18) and to results of 170 MTB cultures was 76.9% (10/13).

**Table 3:** Diagnostic results for bacteriologically-confirmed PTB cases.

	Group	AFB smear	Xpert/ RIF susceptible	Culture	Results of Drug Sensitivity Testing
1	E1	+	+	+	INH^R^
2	R1	+	+	+	PZA^R^
3	R1	+	+	+	INH^R^
4	R1	+	+	+	S
5	EG	+	+	+	INH^R^, RIF^R^, PZA^R^, STM^R^
6	EG	+	+	-	NA
7	EG	+	+	-	NA
8	EG	+	+	-	NA
9	EG	+	+	-	NA
10	E2	+	+	+	S
11	E2	-	+	+	S
12	E2	-	-	+	S
13	R2	+	+	+	S
14	R2	+	+	+	S
15	R2	+	+	+	S*
16	R2	+	+	-	NA
17	R2	+	+	-	NA
18	R2	+	+	-	NA
19	R2	+	+	-	NA
20	R2	+	+	-	NA
21	R2	-	-	+	S*
22	R2	-	-	+	INH*^R^, RIF*^R^

**+** = positive for MTB;— = negative for MTB; **NA** = not applicable

**S** = susceptible to all drugs tested by DST (drug sensitivity testing) or line probe assay (LPA)

* Drug testing by LPA only for INH and RIF

**DST**: INH (isoniazid); RIF (rifampicin); ETH (ethambutol); PZA (pyrazinamide); STM (streptomycin)

^**R**^ = resistant to the indicated anti-TB drug

**Table 4:** Sensitivity and specificity of Xpert for MTB detection.

Xpert									
			Negative		Positive		Total	Sensitivity	Specificity
**AFB Microscopy**									
Negative			719		1		720	100%	99.9%
Positive			0		18		18		
Total			719		19		738		
**Culture**									
Negative			155		9		164	76.9%	94.5%
Positive			3		10		13		
Total			158		19		177		
**BC-TB (excluding Xpert)**									
Negative			785		1		786	85.7%	99.9%
Positive			3		18		21		
Total			788		19		807		

In addition to the 22 BC-TB patients found by symptom screening, 33 prisoners reported being currently treated for TB during their screening, but we do not know how they were diagnosed. Including them more than doubles the prevalence estimate (55/13,803 = 0.40% or 399/10^5^) (Table 2).

*Mycobacterium tuberculosis* (MTB) was isolated by culture in 13 of the 22 BC-PTB cases which were cultured. Drug sensitivity was determined in 10 cases by DST results and in 3 by LPA only. Five of thirteen cases (38%) had MTB drug resistance: two were MDR (one was resistant to INH, RIF, STM and PZA by DST and one to INH and RIF by LPA) and three were mono-DR (two with INH only; one with PZA only). Of the 2 RIF-resistant cases by DST and LPA, one was Xpert negative for MTB and one was MTB-positive, but indeterminate for RIF (Table 3).

### Risk factors for PTB

We compared the 22 BC-PTB cases to those prisoners without BC-PTB for risk factors for PTB (Table 5). Risk factors significantly associated with BC-PTB in the bi-variate analysis included ever having a TB diagnosis (p<0.001), history of TB contact (p = 0.005), never being married (p = 0.028), being underweight (BMI <18.5) (p<0.001) and HIV infection (p = 0.017) and consuming one or more alcohol drinks daily (p = 0.040). Overall, the rate of TB among HIV positive participants in this study was 6.3-fold higher than among participants of negative or unknown HIV status [3/400 (0.75%) versus 12/10,374 (0.12%)].

**Table 5:** Bivariate analysis of BC-PTB and risk factors.

Participant Characteristics	Not PTB n (%)	PTB n (%)	Total (N = 13,803) n (%)	*p-value*
**Gender**			*n = 13*,*803*	***p = 0*.*063***
Male	11801 (99.8)	22 (0.2)	11823 (85.7)	
Female	1980 (100.0)	0 (0.0)	1980 (14.3)	
**Marital Status**			*n = 13*,*803*	***p = 0*.*028***
Never Married	8452 (99.8)	18 (0.2)	8470 (61.4)	
Married	4935 (99.9)	3 (0.1)	4938 (35.8)	
Divorced/Separated	249 (100.0)	0 (0.0)	249 (1.8)	
Widow/Widower	65 (100.0)	0 (0.0)	65 (0.5)	
Other	80 (98.8)	1 (1.2)	81 (0.6)	
**Occupation**			*n = 13*,*803*	***p = 0*.*194***
None	406 (100.0)	0 (0.0)	406 (2.9)	
Student	1485 (99.9)	1 (0.1)	1486 (10.8)	
Housewife	144 (100.0)	0 (0.0)	144 (1.0)	
Merchant	1417 (99.9)	1 (0.1)	1418 (10.3)	
Gov. employee	1096 (99.9)	1 (0.1)	1097 (7.9)	
Peasant	800 (99.5)	4 (0.5)	804 (5.8)	
Other	8433 (99.8)	15 (0.2)	8448 (61.2)	
**TB diagnosis ever**			*n = 13*,*803*	***p<0*.*001***
No	13305 (99.9)	16 (0.1)	13321 (96.5)	
Yes	476 (98.8)	6 (1.2)	482 (3.5)	
**History of TB contact**			*n = 13*,*803*	***p = 0*.*005***
No	13044 (99.9)	17 (0.1)	13061 (94.6)	
Yes	737 (99.3)	5 (0.7)	742 (5.4)	
**Body Mass Index (BMI)**			*n = 13*,*227*	***p<0*.*001***
Underweight (<18.5)	828 (98.9)	9 (1.1)	837 (6.3)	
Normal (18.5–24.9)	10082 (99.9)	12 (0.1)	10094 (76.3)	
Overweight (25–29.9)	2028 (100.0)	1 (0.0)	2029 (15.3)	
Obese (≥30)	267 (100.0)	0 (0.0)	267 (2.0)	
**HIV infection/status**			*n = 10*,*774*	***p = 0*.*017***
Negative	10362 (99.9)	12 (0.1)	10374 (96.3)	
Positive	430 (99.3)	3 (0.7)	433 (4.0)	
**Prison location**			*n = 13*,*803*	***p = 0*.*084***
Entrant	8219 (99.9)	9 (0.1)	8228 (59.6)	
Resident	5562 (99.8)	13 (0.2)	5575 (40.4)	
**Alcohol use prior to prison**			*n = 13*,*794*	***p = 0*.*040***
None to < 1 per day	11,425 (99.9)	14 (0.1)	11,439 (82.9)	
≥ 1 per day	2347 (99.7)	8 (0.3)	2355 (17.1)	

**Variables with p>0.20:** age (p = 0.586), ethnicity (p = 0.709), education (p = 0.745), literacy (p = 0.504), religion (p = 0.559), previous TB treatment (p = 0.410), diabetes diagnosis (p = 0.247), cancer diagnosis (p = 1.000), residence prior prison (p = 0.611), prior imprisonment (p = 1.000), prior prison duration (none = no stat), current prison duration (p = 0.623), current smoking (p = 0.371), current use of Khat (p = 0.913), number of sex partners (p = 0.801), number of people in the house (p = 0.295), and number of rooms in the house (p = 0.862).

Risk factors for BC-PTB identified in other studies such as age, education, occupation, urban/rural residence before prison, prior prison residence, current smoking, current use of Khat, and number of rooms and people in house prior to prison were not significantly associated with BC-PTB (Table 5). In binary logistic regression analyses, ever having a TB diagnosis (p = 0.005), history of TB contact (p = 0.047) and Body Mass Index (BMI), (p = 0.047) were significantly associated with BC-PTB. Having a BMI that is normal (AOR = 0.196, p = 0.009) or overweight (AOR = 0.099, p = 0.047) was protective (Table 6).

**Table 6:** Multi-variate analysis of BC-PTB infection and its related risk factors.

Characteristic/Variable	AOR	95% C.I.		p-value
		Lower	Upper	
**Gender**				
Male	1.55 x10^6^	0.000	-	**0.987**
Female	1	-	-	-
**Marital Status**				**0.273**
Never Married	1	-	-	-
Married	0.361	0.097	1.34	0.128
Divorce/Separated	0.000	0.000	-	0.995
Widow/Widower	0.000	0.000	-	0.997
Other	5.99	0.554	64.7	0.141
**Occupation**				**0.532**
None	1	-	-	-
Student	0.898	0.000	-	1.000
House Wife	11. 3	0.000	-	0.999
Merchant	1.89 x10^6^	0.000	-	0.994
Government Employee	2.66 x10^6^	0.000	-	0.994
Peasant	1.08 x10^7^	0.000	-	0.994
Other	2.46 x10^6^	0.000	-	0.994
**TB diagnosis ever**				
No	1	-	-	-
Yes	6.42	1.745	23.6	**0.005**
**History of TB contact**				
No	1	-	-	-
Yes	3.94	1.02	15.2	**0.047**
**BMI (Body Mass Index)**				**0.047**
Underweight	1	-	-	-
Normal weight	0.196	0.058	0.66	0.009
Overweight	0.099	0.010	0.97	0.047
Obese	0.000	0.000	-	0.995
**HIV infection/status**				
Negative	1	-	-	-
Positive	3.20	0.739	13.8	**0.120**
**Alcohol use prior to prison**				
None to < 1 per day	1	-	-	-
≥ 1 per day	1.14	0.351	3.70	**0.828**
**Prison location**				
Entrant	1	-	-	-
Resident	1.06	0.333	3.34	**0.927**

*AOR = Adjusted Odds Ratio

**95% C.I.** = 95% Confidence Interval

## Discussion

Through an enhanced PTB screening program for entrant and resident prisoners in a large Ethiopian prison, we found an unexpected low overall prevalence of bacteriologically-confirmed pulmonary tuberculosis (BC-PTB, 0.16%). Although higher than in the entrant prisoners, the prevalence of BC-PTB (0.23%) in the resident population was lower than that found in 4 of 5 studies of residents in other Ethiopian prisons that used similar diagnostics (mean 0.67%; range 0.065–1.5%) **[15–18]**.

The reasons for this overall low prevalence are unclear, but multiple factors may have contributed. First, we did not include the prisoners who had been diagnosed by screening or routine care before our screening began. Second, a TB prevention campaign conducted by Ethiopian Federal Ministry of Health that had reduced TB prevalence in the general population might have contributed to lower rates. Third, better-organized and -resourced medical care implemented at Kality prison in the years before our study may have reduced intramural TB transmission. Fourth, we did not include those prisoners with TB identified during screening by routine care. Fifth, collection of and processing sputum for AFB microscopy dramatically increased due to implementation of the screening program prior to what the prison laboratory had previously performed. Sixth, an evaluation of sputum quality showed that the majority of all samples collected were classified as saliva, as opposed to mucopurulent or purulent, with 88% (1,452/1,649) saliva from the first sample. Finaly, the yield of AFB smear positive cases from saliva was <0.5% (0.3%, 5/1,452) compared to mucopurulent (up to 7%, 9/129) and purulent (up to 3%, 2/68) samples. Thus, the quality of sputum induction in addition to sample processing and AFB smear analysis, may have been inadequate resulting in fewer positive BC-PTB cases.

Our participants were demographically similar to participants in prior prison studies in Ethiopia, i.e. consisting primarily of young unmarried men who were educated to less than secondary levels, but literate and without prior incarcerations **[11,15–18]**. Thus, i the demographic characteristics of Kality Prison population were similar to those of other PTB prevalence studies and thus appears unlikely to have accounted for our findings.

One-third (35%) of resident prisoners reported at least one PTB symptom, a prevalence rate about twice that reported in 3 of 5 prior studies examining TB symptoms in Ethiopian prisoners (14.6–19.4%) **[11,17,18]** and about 7–10 fold higher than rates found in the 2 other studies (3.4% and 4.9%) **[15,16]**. In addition, screening symptoms for TB were reported at a seven-fold higher rate in residents than entrants. The ratio of patients with BC-PTB to those reporting possible symptoms of PTB in both groups of prisoners was very low (< 1%). Thus, the low prevalence rate of PTB compared to the high rate of screening symptoms in both groups of prisoners indicates that most reported symptoms in both groups were likely unrelated to TB.

The lower prevalence of BC-PTB in entrants compared to residents was expected. This finding supports incident intramural transmission and/or of reactivation by resident prisoners of latent PTB acquired pre-incarceration by resident prisoners **[19]**. Stressful conditions in prisons such as crowding, poor ventilation, extremes of temperature, and inadequate nutrition could increase both transmission and reactivation **[19–21]**. Our study could not address which of these mechanisms could have influenced prevalence of PTB because we had no access to the interior areas of the prison or to records of the census data (admissions, transfers, and discharges) that could change intensity of crowding.

The nearly four-fold higher prevalence in the second of our two resident surveys (0.39%) than in the first (0.10%) contradicts our hypothesis that identification, isolation, and treatment of entrants and residents with PTB during the first wave of screening would reduce the prevalence in the second wave in residents. The reason for this finding is unclear.

The estimated national prevalence of multiple drug resistant (MDR) MTB is 2.7% in new cases and 14% in previously treated cases **[22]**. Of the 10 strains of MTB isolated in culture and tested for drug resistance in liquid culture, 5 (38%) were drug resistant (2 were MDR and 3 were mono-DR). While neither liquid culture nor Xpert dramatically increased diagnostic sensitivity compared to AFB microscopy, culture with DST or LPA detected drug resistance in 38% of cases. Xpert, which detects mutations associated with rifampicin resistance in a day rather than up to 2 months that is required for culture-based methods, did not contribute to finding suspect MDR TB cases in this study.

While we found a substantial prevalence of MDR-PTB in those tested (2/13 = 15%), the small numbers of cases tested limits our confidence in the accuracy of this estimate. Institution of drug susceptibility testing during screening with liquid culture is expensive, slow, and probably not a high priority for the prison. However, improvement in the sensitivity of Xpert assay might allow for better detection of rifampin resistance with a greater focus on these isolates. Drug susceptibility testing was not reported in the previous five prison studies in Ethiopia **[11,15–18]**.

Variable results of sensitivity and specificity of GeneXpert have been reported by various studies. A similar finding of sensitivity (79.7%) and specificity (98.2%) of GeneXpert using culture as the gold standard was reported by a hospital based study in Turkey **[23]**. Contrary to our findings, GeneXpert had a higher sensitivity (100%) and specificity (99.4%) relative to culture in a study at a county hospital in Kenya **[24]**. This relatively low sensitivity of Xpert in our study could be due to the inherent low detection rate of mycobacterial culture compared to GeneXpert in detecting MTB from salivary sputum specimens **[25]** because 88% of our samples were classified as saliva. Sensitivity of the GeneXpert with smear and culture-positive pulmonary specimens, equivalent to the “BC-TB excluding GeneXpert” in our study, in the Turkish hospital based study was higher (100%), but the specificity was similar (98.3%) **[23]** compared to our study.

In this and previous studies, HIV infection increased the risk of PTB regardless of the stage of HIV disease stage or treatment success achieved with antiretroviral drugs **[26]**. Risk of TB decreases after treatment of HIV with ART by increasing immunocompetence **[27]**. Thus, expanding access to ART in the populations both in and out of prison should decrease TB prevalence in both entrants and residents. However, among the 22 prisoners with BC-PTB, only 3 (14%) were HIV-infected. This HIV prevalence among PTB patients diagnosed primarily by AFB smears is less than half of that in a study from a regional Ethiopian prison in the North Gondar Zone using the same methods (35%). This finding may reflect declining PTB incidence rates in HIV patients resulting from rapid expansion of antiretroviral drug coverage in Ethiopia **[28,29]**.

Risk factors for BC-PTB, after controlling for other factors included ever having TB, history of TB contact, and being underweight. As in our study, low BMI (<18.5) was associated with TB in the Wolaita and Gondar Zone prisons studies **[17,18]**. Causal relationships among low BMI, HIV and TB are potentially bidirectional because both HIV and low BMI are risk factors for TB and HIV is risk factor for low BMI. Other risk factors for TB described in previous studies (e.g., age, occupation, residence before prison, smoking, etc.) were not significantly associated with PTB in our study **[11,15–18]**.

We found no link between smoking and TB in spite of evidence from many studies of a strong relationship. The relatively low rate of current smoking in these prisoners (16.6%) reduced our power to detect a relationship. A 14-year prospective cohort study (1992–2006) of 1,294,504 South Koreans, found that current male smokers had greater risk of incident tuberculosis than former smokers (HR = 1.4, 95% CI: 1.3, 1.5), and risk among current smokers increased with number of cigarettes smoked daily **[30]**. Review of 34 studies of the association between smoking and tuberculosis reported that smoking (both current and former) is associated with risk of being infected with and dying of tuberculosis with a strong dose-response relationship for both quantity and duration of smoking **[31]**.

Our study also has multiple limitations. First, the sensitivity of sputum examinations was compromised by a high percentage of inadequate sputum specimens (reported as saliva). Second, we could not evaluate recruitment and interview methods due to lack of access to areas of high security by study staff other than prison health care workers. Third, we were unable to include prison guards and other staff who share the environment with prisoners and may be sources of infection. Fourth, some potential participants were missed due to refusals, after hour admissions and occasional surges of entrants that overwhelmed capacity of the staffto recruit. Fifth, of the prisoners who did not participate in the study, only 25 completed refusal surveys, and thus reasons for refusal were not adequately captured. Sixth, prisoners transferred from other federal prisons primarily for PTB were placed directly in medical facilities and thus not screened through the study. Seventh, the screening questionnaire was subject to recall bias that could result in prisoners over-reporting risk factors. Finally, our estimates of prevalence and analysis of risk factors associated with PTB was limited by the small number of bacteriologically-confirmed TB cases.

Strengths of our study included its cross-sectional screening of large numbers of both residents and entrants to the prison, utilization of multiple diagnostic methods for TB, and quality control of prison-based laboratory testing by an internationally-accredited laboratory.

## Conclusions

Despite a high prevalence of possible PTB symptoms in our participants, BC-PTB and TB/HIV co-infection cases were unexpectedly low compared to reports from smaller prisons in Ethiopia. Prevalence of BC-PTB in residents increased rather than decreased in the second compared to the first wave of screening, contrary to our study hypothesis that continued screening would decrease prevalence. Prevalence of BC-PTB in resident prisoners was double that in entrants to the prison, supporting intramural transmission and/or increased rates of reactivation of latent PTB in the prison.

Since most cases of BC-PTB in our participants were detected by sputum microscopy (18/22 = 82%) and only 4 were found by culture only, the cost of adding cultures to screening was high per case detected. The low rate of undiagnosed BC-PTB detected in entrants may suggest that they should not be the first priority for screening. The high cost and low prevalence of MDR PTB found by screening lowers the priority for including universal culture-based diagnosis and drug sensitivity testing for screening in this resource-limited setting. However, detection of multi- and mono-DR TB in both entrant and resident prisoners warrants regular screening for active TB in prisons if resources permit. Whether a sustained program of screening residents reduces prevalence of PTB in prisons is not clear from this study, the question deserves further study.

## Supporting information

S1 File Code books. (XLSX)

S2 File Data (with codes). (XLSX)
